# User interface design for mobile-based sexual health interventions for young people: Design recommendations from a qualitative study on an online Chlamydia clinical care pathway

**DOI:** 10.1186/s12911-015-0197-8

**Published:** 2015-08-26

**Authors:** Voula Gkatzidou, Kate Hone, Lorna Sutcliffe, Jo Gibbs, Syed Tariq Sadiq, Ala Szczepura, Pam Sonnenberg, Claudia Estcourt

**Affiliations:** Department of Computer Science, Brunel University, Uxbridge, UK; Barts and the London School of Medicine & Dentistry, Queen Mary University of London, London, UK; Institute for Infection and Immunity, St. Georges, University of London, London, UK; Centre for Technology Enabled Health Research, Coventry University, Coventry, UK; Research Department of Infection and Population Health, Mortimer Market Centre, University College London, London, UK

## Abstract

**Background:**

The increasing pervasiveness of mobile technologies has given potential to transform healthcare by facilitating clinical management using software applications. These technologies may provide valuable tools in sexual health care and potentially overcome existing practical and cultural barriers to routine testing for sexually transmitted infections. In order to inform the design of a mobile health application for STIs that supports self-testing and self-management by linking diagnosis with online care pathways, we aimed to identify the dimensions and range of preferences for user interface design features among young people.

**Methods:**

Nine focus group discussions were conducted (n = 49) with two age-stratified samples (16 to 18 and 19 to 24 year olds) of young people from Further Education colleges and Higher Education establishments. Discussions explored young people’s views with regard to: the software interface; the presentation of information; and the ordering of interaction steps. Discussions were audio recorded and transcribed verbatim. Interview transcripts were analysed using thematic analysis.

**Results:**

Four over-arching themes emerged: privacy and security; credibility; user journey support; and the task-technology-context fit. From these themes, 20 user interface design recommendations for mobile health applications are proposed. For participants, although privacy was a major concern, security was not perceived as a major potential barrier as participants were generally unaware of potential security threats and inherently trusted new technology. Customisation also emerged as a key design preference to increase attractiveness and acceptability.

**Conclusions:**

Considerable effort should be focused on designing healthcare applications from the patient’s perspective to maximise acceptability. The design recommendations proposed in this paper provide a valuable point of reference for the health design community to inform development of mobile–based health interventions for the diagnosis and treatment of a number of other conditions for this target group, while stimulating conversation across multidisciplinary communities.

**Electronic supplementary material:**

The online version of this article (doi:10.1186/s12911-015-0197-8) contains supplementary material, which is available to authorized users.

## Background

Sexually transmitted infections (STIs) are a major public health issue with important and costly personal and population health consequences [1]. Young people are disproportionately affected [2] and people under 25 years account for almost half the annual reported cases of *Chlamydia trachomatis*, the commonest STI. Although the proportion of young people testing for chlamydia is increasing [3] significant practical and cultural barriers to engaging young people in routine testing for STIs remain [4, 5, 6] and there is considerable scope to widen access to STI testing and care. The explosion of technology presents new opportunities to provide online sexual health services irrespective of gender, age, sexual orientation and location [7]. The ubiquity of mobile phones combined with their increasing communication capabilities present an opportunity for effectively addressing the individual and social barriers that limit the uptake of testing for STIs among young people. However, the design of such interventions needs to be carefully planned and evaluated in order to ensure that the interaction is both usable and acceptable to potential users. The user interface is a key consideration as it provides the user with both the means to reach their interaction goals with the system and their main insight into the nature of the wider healthcare system with which they are interacting.

Mobile health (m-health) innovations have the potential to address a number of contemporary healthcare concerns such as increased demands for personalisation of care, disease prevention, expectations of health care provision and threat of pandemics. Mobile phone penetration worldwide is growing at an increasing pace (7 billion subscriptions as of October 2013) with 79 % of 18–29 year olds using mobile apps daily [8]. There are currently more than 97,000 m-Health applications listed on 62 full catalogue app stores [9]. Extensive reviews of the use of mobile phone and handheld computing devices in health and clinical practice can be found in the literature [10, 11]. Currently, sexual health interventions have turned towards internet based education [12] ; STI screening, testing and management [13, 14] including partner notification [15].

Mobile- specific sexual health interventions are also being used for the prevention and care of STIs, with initiatives in both the developed and developing world, primarily focusing on promoting prevention messages [16], facilitating test result notification [17] and increasing adherence to clinic appointments [18].

None to date have included clinical consultations for people with a new STI diagnosis, leading to electronic antibiotic prescribing. However, the evidence-base on feasibility and user preferences for mobile health applications is relatively limited and nascent [19] and there exists little data discussing how young people make use of Web and mobile technology and its impact on their sexual health care [20].

The specific focus of the work described here was the design of a mobile application via which patients can access the results of STI self-testing in the community, complete an online medical assessment and (for those whom it is safe to treat) receive access to treatment via an electronic prescription. The system would also facilitate partner notification, the process by which exposed sex partners of people with STIs are identified, tested and treated [21] by enabling partners to access the same system remotely from traditional health settings via their mobile phone [22].

While a number of user interface guidelines were found in the literature relevant to the design of mobile health interventions for chronic conditions [23–27] (Additional file 1) there is a dearth of relevant empirical findings in the context of infectious diseases and specifically for the kind of novel STI intervention considered here. While these recommendations provide a useful starting point, it is unclear to what extent these will also apply to health applications targeting more acute conditions or those which actually test for an infection and are capable of providing a user with a new diagnosis of an acute STI without any contact with a traditional service. It is likely that additional features may be of concern in such applications, particular for sexual health, where issues of privacy are likely to be particularly salient.

Furthermore, even though the market is seeing exponential growth in the number of health-related applications available for mobile devices, quality is a concern. In the field of sexual health related apps, recent research suggests that these are infrequently downloaded and not highly rated by users [20]. Careful design to address a better fit between technological, human and contextual factors is essential to the uptake and impact of mobile health technologies. This requires a good understanding of the requirements of the potential end users of the technology. In order to identify interface design requirements for this novel intervention, exploratory research using focus groups was therefore conducted with young people from the age categories most at risk of acquiring STIs. Here we present our thematic analysis of the resulting discussions to identify users’ functional and non-functional user interface design requirements and propose design recommendations applicable to mobile sexual health application user interface design.

## Methods

Focus groups were chosen to elicit user interface design requirements as they represent a suitable method of exploring design spaces and concepts [28, 29]. The choice of this particular method was due to the fact that we wanted to explore the dynamics created by participants in a peer setting where participants are more likely to share experiences, stories, memories, perceptions, wants and needs. Despite the sensitive nature of the discussion topic, people may be more willing to talk openly about issues of sensitive nature like sexually transmitted infections when in a group of people with similar experience than they would be in a one to one interview [30]. The group dynamics can provide deep insights into themes, patterns and trends, which makes focus groups particularly useful in exploring shared meaning at exploratory stages of a study [31]. As the proposed STI intervention under consideration was highly novel, the focus group design made use of experience prototypes [32] to help the participants conceptualise the nature of the intervention and facilitate discussion.

The Research Ethics Committee of Brunel University London reviewed and approved the focus group protocols and ethical approval had been granted prior to the study.

### Sampling and recruitment strategy

The study took place in a Higher Education (HE) Institution in London and a Further Education (FE) College in an economically disadvantaged area in the North East of England where there are high rates of chlamydia diagnoses [2]. In order to explore a diverse set of preferences, attitudes and perspectives, the inclusion criteria for the study were intentionally relaxed and were only age (16 to 24 years old) and smartphone ownership.

In both settings, participants were recruited using convenience sampling methods. In the HE setting, the opportunity to take part in the research study was advertised through the internal website, and participants who met the inclusion criteria were sent further information about the study via e-mail. In the FE setting the researchers contacted the staff at the college and agreed on the method for approaching the participants where college staff invited students to participate in the study. College staff would organise and arrange the discussions for the participants who met the inclusion criteria.

### Focus group format

Focus groups were conducted in 2013 with samples from two groups of mobile phone users: 16–18 and 19–24 year olds; age groups which are representative of potential users with the highest risk of STI infection. Discussions where conducted in a private room at the FE/HE college campus, lasted for 45–60 minutes and were audio recorded and facilitated by the lead researcher. Participants had the option to select participation in same sex or mixed-sex group discussions. On arrival, information sheets providing the context, purpose of the study and a summary of activities, were distributed to participants, the content of which was worked through with each participant. Informed consent was obtained and participants were then asked to complete a short questionnaire on their demographics.

A semi-structured topic guide was used to promote discussion of the content and functionality of the application (Additional file 2). This covered the feasibility, acceptability, and attractiveness of potential features of the mobile application being proposed as well as visual design, information architecture, structure organisation, labelling of visual components, finding and managing options and interaction design.

**Fig. 1:**
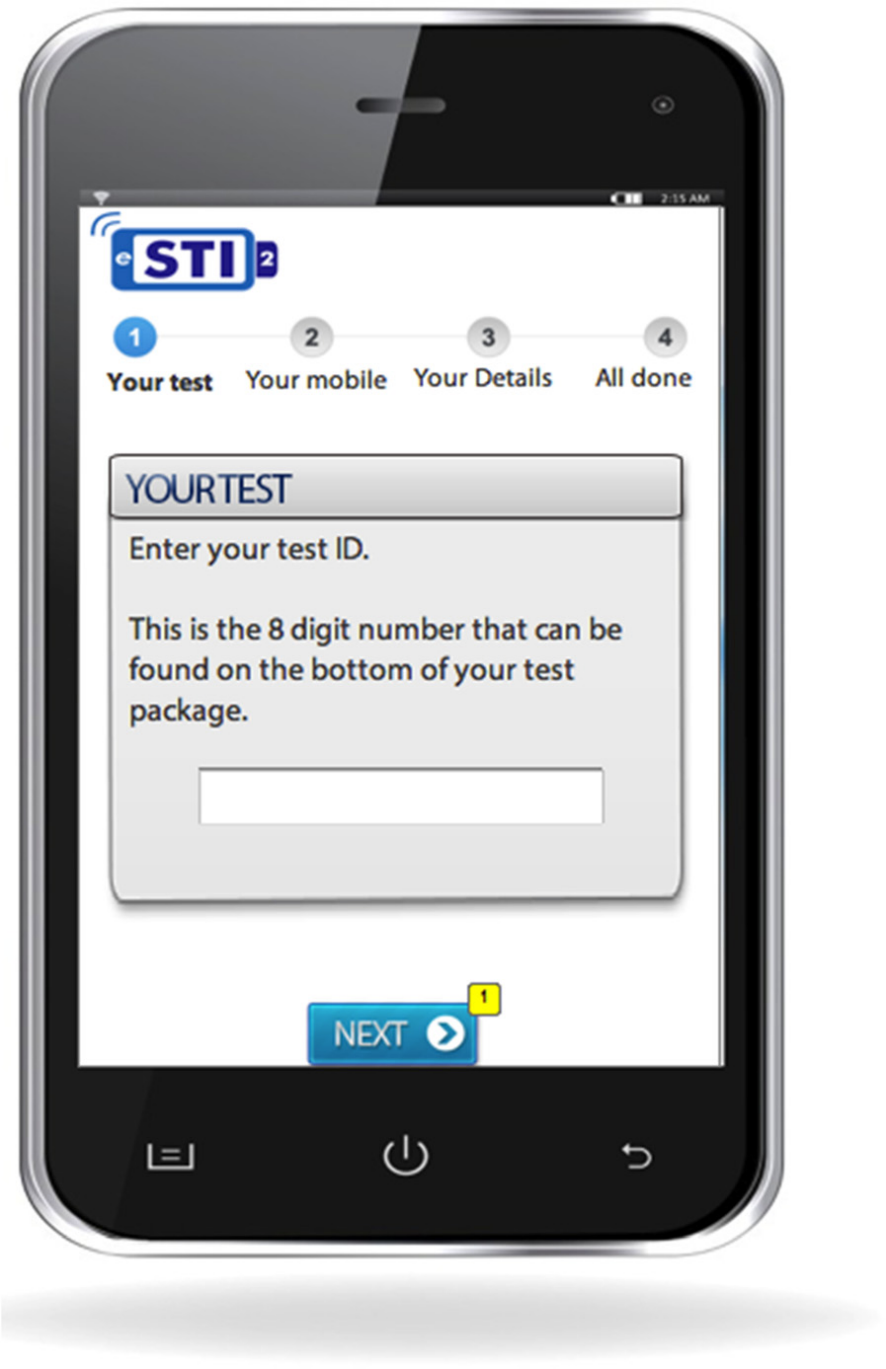
Application prototype used in focus group discussions (login screen)

**Fig. 2:**
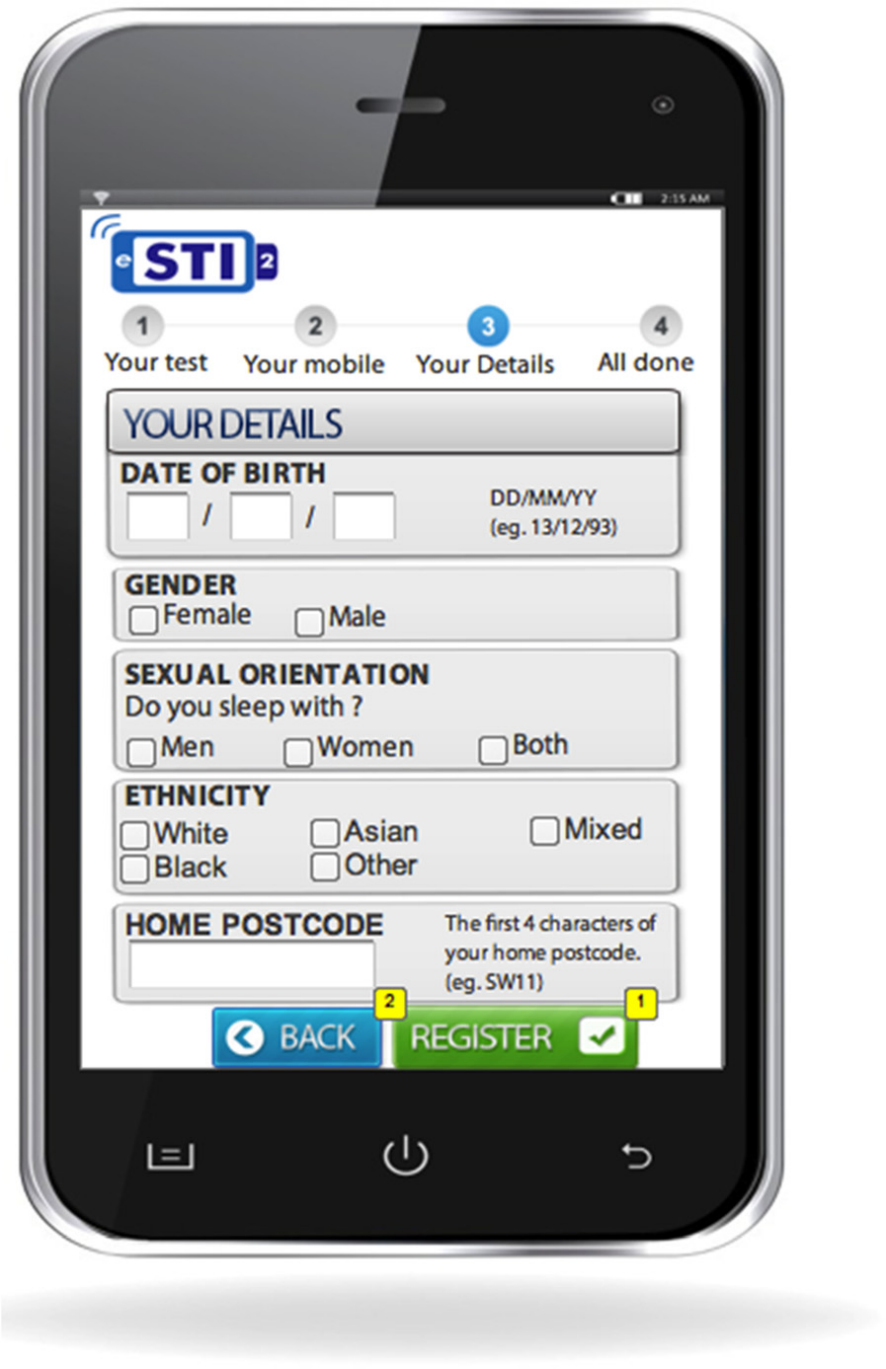
Application prototype used in focus group discussions (Registration screen)

**Fig. 3:**
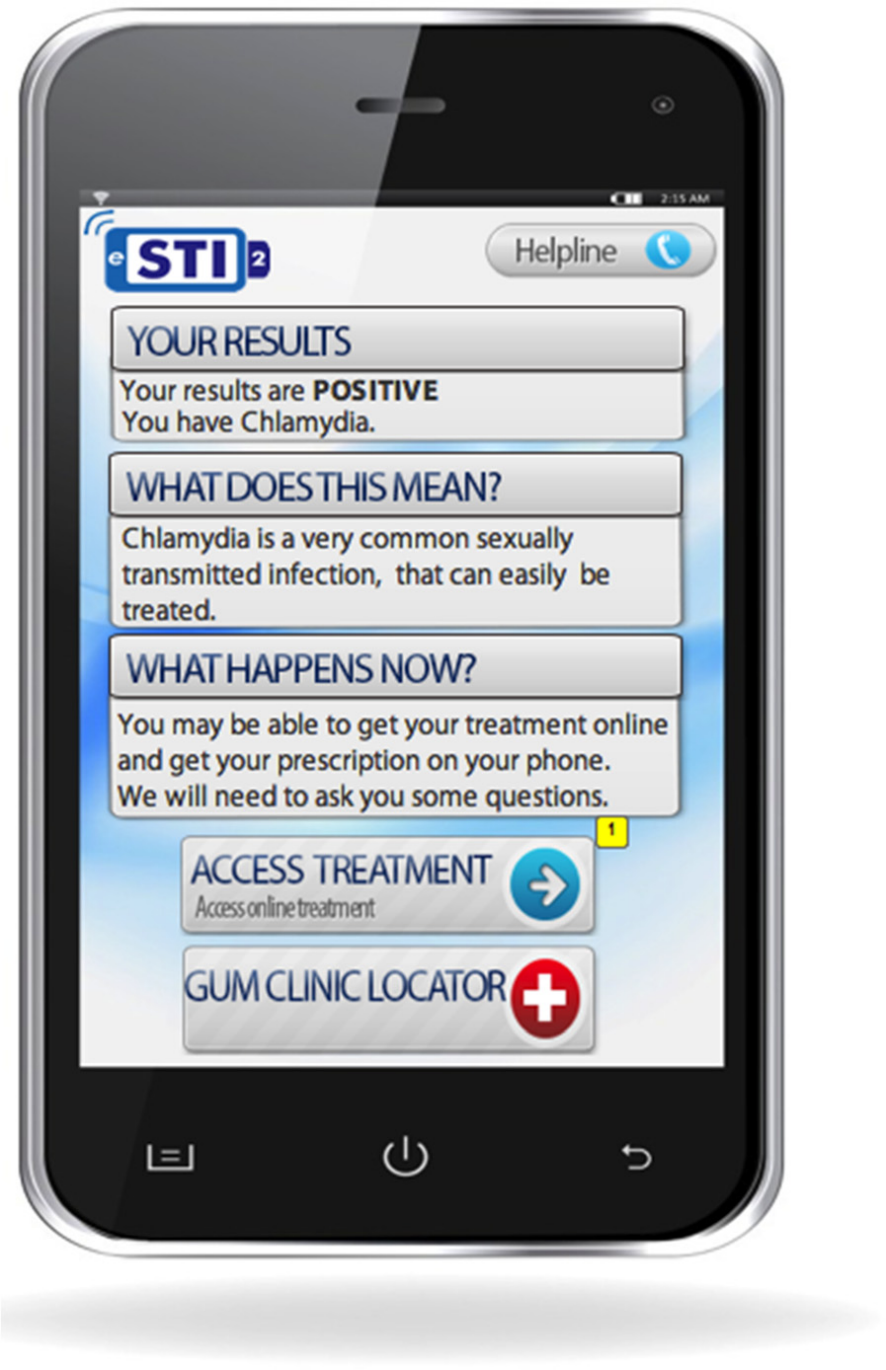
Application prototype used in focus group discussions (medical consultation screen

A low fidelity prototype of the sexual health application was developed through an iterative and cross-disciplinary reviewing process, exploring design possibilities for message content, modality and delivery platform in order to provide a prompt for discussions. This was also informed by a preliminary qualitative interview study to explore young people’s perceptions of the concept of using electronic self-tests for STIs linked to mobile technology for diagnosis and care [33]. The prototype mobile application (Figs. 1, 2, 3) was developed using Axure PR software and the interface was developed enough to allow exploration of the system. The focus group facilitator demonstrated the prototype application on a laptop screen.

**Fig. 4:**
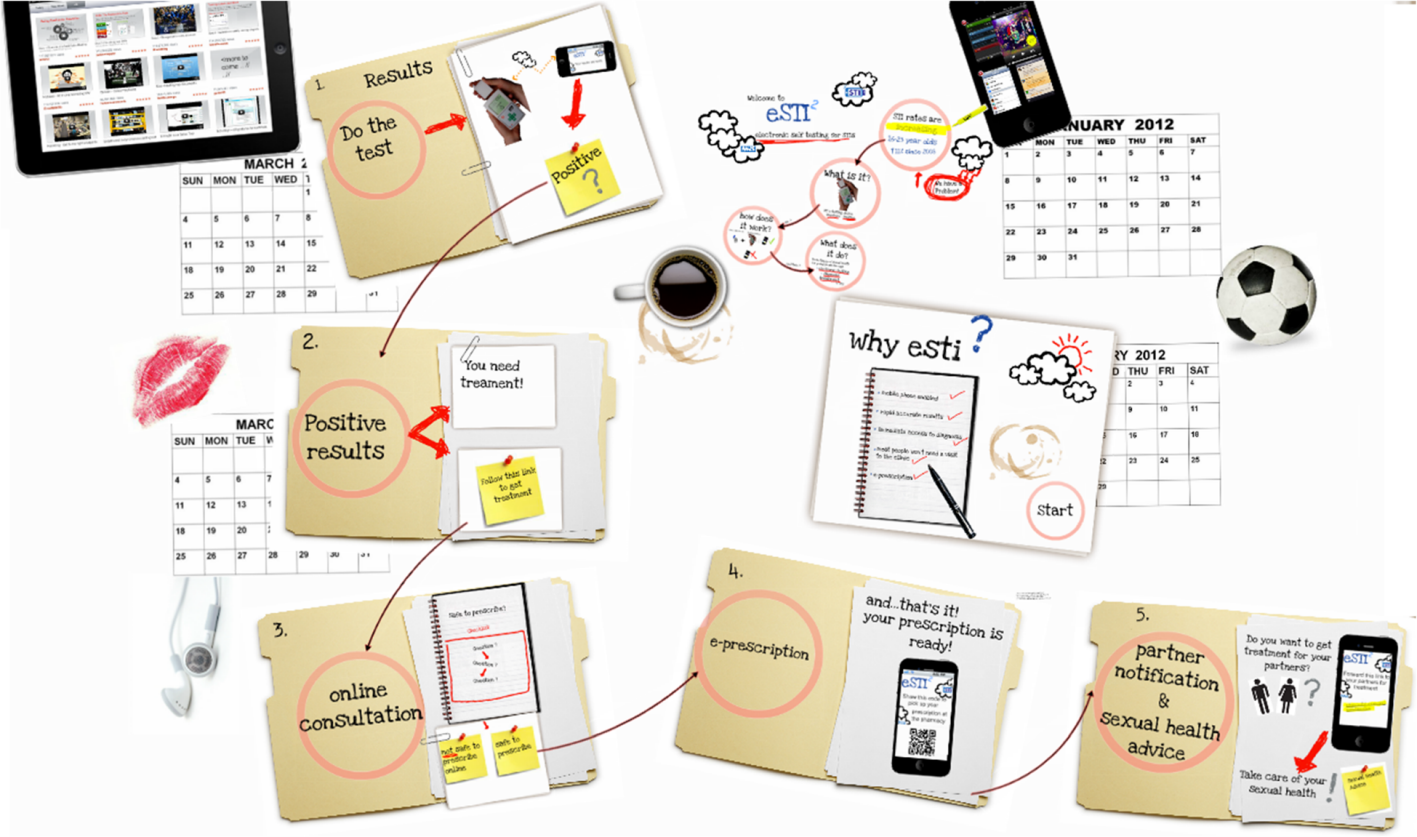
Visual probe animation of underlying clinical pathway

In addition, an animation of the underlying clinical pathway (visual probe) of the system was developed using Prezi (Fig. 4). The aim of the visual probe was to ensure that all participants, regardless of previous experience with face-to-face STI testing and consultation, would understand the main steps involved in the process.

The animation of the underlying clinical pathway Fig. 2) was used at the beginning of the focus group sessions to set the context of the discussion and engage the participants. The prototype (Fig. 1) was also presented early in the session to engage young people in discussions about their views in regard to the interface, how the information is presented and the ordering of interaction steps. Participants were asked to imagine providing a urine sample at home, undergoing a self-test, similar to a pregnancy test but in which the results are only available on their mobile phone. The eSTI^2^ mobile app was presented to users on–screen and they were asked to interact with a number of use case scenarios. Scenarios describe a sequence of actions users will try to do when they use a system, ensuring that design will remain focused on the needs and concerns of users [34]. (An example of a scenario can be found in Additional file 3).

### Data Analysis

Audio recordings of the discussions were transcribed verbatim and thematic analysis of the textual dataset was carried out by two members of the research team. Given the exploratory nature of the work, coding was conducted inductively rather than being driven by *a priori* themes from the literature, [27]. This particular method has been widely applied within the context of HCI, to inform the design of new technology interfaces [35, 36], identify key interaction challenges by analyzing users experiences with technology prototypes, [37] and define the functionality of new technology [38]. The ‘Framework’ approach was used [39] to analyse the data, where data from transcripts s coded, indexed and charted systematically and analysis is conducted deductively from the study aims and objectives, but is also inductive (reflecting the original account and observations of the people studied). Key issues, concepts and themes are identified by drawing on a priori issues and questions derived from the topic guide as well as issues raised by the respondents themselves and views and experiences that recur in the data. Themes were identified which integrated substantial sets of the codings, mapped and interpreted. The author and a co-author (KH) undertook the analysis and reliability was enhanced by double coding and comparing a subset of transcripts with other two co-authors (JG, LS). Few discrepancies emerged and, where they did, consensus was negotiated. Qualitative data analysis software (QSR NVIVO 10) was used to frame key topics and code the overarching themes that existed within the transcripts at a high level. These were noted in a coding frame with each concept assigned a code name, description and examples of text that fit each concept. The next step of the analysis involved identifying a list of high-priority themes and sub-themes against which design recommendations could then be formulated. This was achieved through a group discussion and consensus building process (VG, KH), which provides a method for synthesising a range of information [40] whilst harnessing the insights of multi-disciplinary researchers involved in the project.

## Results

### Participants

Overall, 49 participants (n =49) took part in in nine focus group discussions- three female-only, two male-only, and four mixed sex groups (Table 1). Median age of participants was 19 years, 29/49 (53 %) were female and 32/49 (65 %) were of white ethnicity. Participants were recruited from a Higher Education Institution (49 %) in London and a Further Education College in the North East of England (51 %)

**Table 1:** Focus group participant demographics

Group	Male	Female	Age group	Location
1	4 male	2 female	Over 18	HE
2	1 male	4 female	Over 18	HE
3	-	5 female	Over 18	HE
4	4 male	-	Over 18	HE
5	4 male	3 female	Under 18	FE
6	3 male	3 female	Under 18	FE
7	-	5 female	Under 18	FE
8	-	5 female	Under 18	FE
9	6 male	-	Under 18	FE

.

During the sessions, participants expressed general enthusiasm towards the concept of self-managing their sexual health through a mobile application. The four key interface design requirement themes which emerged were: privacy and security, credibility and legitimacy, user journey support and task-technology-context fit. Within these broad categories a number of sub-themes also emerged. The sections that follow describe the results in more detail with illustrative examples from participants’ comments.

### Theme 1: Privacy and security

Privacy was the greatest concern. Participants were primarily concerned with their ‘social’ privacy when using the application rather than ‘institutional’ privacy, expressing concerns about controlling access to personal information on their phone itself, particularly by friends and family.

*‘I live in halls and you know how it is, people just constantly grab your phone off you to check what games and apps you got… I have a passcode on my phone, but that is like 4 digits, my mates already know it anyway’. [Participant 33, Male, age 18]*

Participants consistently voiced privacy concerns with regard to system notifications, predominately surrounding test results and partner notification. Participants felt that the choice of modality of notifications received from the app could pose a privacy threat to them. For example participants argued that a results notification delivered as a text message could potentially be embarrassing in a social setting, and preferred to receive it as an in-app notification (icon only, no audio alert) which they can control and personalise through the mobile phone’s notification settings. The 19–24 year old age group preferred to receive and store all the notifications within a dedicated application, rather than on email or text, while this appeared to be less of an issue for the younger group (16–18).

*‘I wouldn’t want a text from the app….because my phone can beep and then anyone can just grab it and check. An app notification would be better … I would know what the update is about but no one else would.’* [Participant 4, Male, age 21] *‘…if I tested for an STI and I was waiting for my results, I would be careful of where I leave my phone and who is around me.’* [Participant 45, Female, age 16]

Privacy concerns also spanned the design of the interface including the application’s name and logo design.

*‘I grew up in a Christian family…and this is a ‘hot topic’ …. I wouldn’t want my sister, or my mum or my dad finding an app on my phone that says sexual.’* [Participant 31, Female, age 23]

While less salient than ‘social’ privacy, some ‘institutional’ privacy concerns did emerge. Even though participants recognised the value of ‘registering’ with the service through the app, which implies the disclosure of personal data, concerns were raised about who would have access to their data.

*‘I am quite careful about where I put my data online, as soon as one of these companies gets a piece of information, it just goes to everybody and then the next day you get 50 emails or texts or whatever.’* [Participant 11, Female, age 22]

Occasionally participants became frustrated when they were asked to disclose certain information about themselves, especially when they felt this was ‘sensitive’ information (e.g. postcode or sexual history information).

*‘Why are you asking for postcode? Will you send me leaflets?…Full postcode narrows it down to a street, so if you are in the middle of nowhere and there are no families living around there, within the range they could trace it back to you.’* [Participant 8, Female, age 18]

There also seemed to be concerns that stored electronic information was more likely to be accessed by unauthorised third party organisations. However, in general security was not deemed to be a major barrier to use, with participants inherently trusting new technology and being unaware of a number of potential security threats.

### Theme 2: Credibility & Legitimacy

Credibility in the context of an online interaction is defined as the initial judgments based on surface traits, which in the context of an online interaction is based on the ‘look and feel’, aesthetics or design of a web interface [36–38]. Credibility was a concern to the majority of the participants who saw the design and the content of the application as cues to determine the credibility of the service. The participants used the terms ‘credibility’ and ‘legitimacy’ interchangeably. Concerns were raised over the credibility of the overall service, especially in relation to the provision of electronic prescription and the legitimacy of medical content.

*‘To be honest, I would think that you are just asking this stuff so you get all this information about me and then it will say ‘sorry but we can’t prescribe you online’. I don’t think the app can do prescriptions online’. I wouldn’t trust it.’* [Participant 27, Female, age 18]

Trust was frequently mentioned in relation to credibility and a number of references were made to the expertise of those responsible for authorising the prescription online.

*‘If I was using this and it just told me I have got Chlamydia but I can get treatment online, I would be a bit suspicious. How do I know that this medication that they are prescribing me is the right one… and WHO is this person prescribing me?’* [Participant 4, Male, age 21]

Discussion also highlighted a number of attributes and cues which ameliorated participants’ credibility concerns. Both non-verbal interface cues (such as colour) and verbal cues (e.g. tone of language) influenced participants’ views of credibility. For example, the following quote illustrates the role of visual design.

*‘I like the logo colours, because green and blue are health-related colours, pharmacy signs are green and the NHS logo is blue, so I think it looks serious.’* [Participant 34, Female, age 16]

Language was also deemed as an indication of the credibility of the medical content of the app. Parallels were drawn to a face-to-face experience of receiving the diagnosis in clinical settings.

*‘I like that it’s serious but it doesn’t sound scary-It says you have chlamydia but you can easily treat it. Sounds like something a doctor would say.’* [Participant 9, Female, age 19]

As well as the app appearance, participants suggested that potential credibility concerns could be ameliorated when the app becomes widely available distributed and marketed.

*‘I mean this is totally new, so you would think twice before trusting it. If I saw it advertised somewhere, or available in Boots then I would think it is …you know…legit’. [Participant 14, Male, age 18]*

An accompanying website for the app and social media presence (i.e. Facebook page) were also mentioned as cues to determine the credibility of the service.

*‘I mean this is totally new, so you would think twice before trusting it. If I saw it advertised somewhere, or available in Boots then I would think it is …you know…legit’. [Participant 14, Male, age 18]*

### Theme 3: User journey support

This theme encompasses the clarity of the interaction with the system from the user perspective (both in terms of knowing where they are in the interaction overall and understanding what data input is required at each point) and extent to which users perceive they can receive help and assistance to successfully complete the task if needed. The following comments made during discussion of the low fidelity prototype (focus group probe) illustrate the potential for participants to get lost within an interaction and be confused over the ordering of steps in the interaction process.

*‘Can I just ask where we are in the progress at this moment? When will I be shown this? Is this just after I picked up the testing device? Where will I be when I get to this menu? Where do I go first, do I go to the app first or do I go to the self-testing device’. [Participant 28, Female, age 17]*

*‘So do I get the app first and register and then do the test?’ [Participant 29, Male, age 22]*

Participants also faced difficulties understanding how to respond to some specific types of questions, for example questions which asked about symptoms were particularly problematic.

*‘I am worried that people might have something completely unrelated, like ‘rash’; some people have eczema, so they might be worried. So it is assuming that it means a rash….well…’down there’…but maybe it actually should specify’. [Participant 7, Female, age 20]*

Even though the prototype mobile interface included contact telephone information in every page, participants were not clear on and did not necessarily understand the nature of the help provided.

*‘It’s good that there is a helpline there and wouldn’t have an issue of calling up and asking for help if I had any questions, but not if it is like a call centre-I mean I would rather ask my mum, she knows more about these things’. [Participant 41, Female, age 16]*

Overall, the consensus was that further support is required to aid and guide the user through a novel mobile based health intervention.

*‘I get it now, and if I had to do it again it would be dead easy but it seems a bit confusing the first time. Maybe you can include a step-by-step guide, for the people who are using it for the first time’. [Participant 47, Female, age 17]*

### Theme Four: Task-technology-context fit

The final theme to emerge from the focus group discussions concerned the three-way fit between the technology, the task and the context of use. Mobility and ubiquity were both identified as key technology attributes. While participants agreed they would access the service on a mobile device, they were also prepared to adopt a flexible and fluid approach towards accessing the service on other platforms. Choices reflected the perceived characteristics of the different platforms and the fit with task attributes. For example:

‘*…I would get my results on my phone, and if I had it [Chlamydia] then I would do the whole thing on my phone because no one has access to that. But I would check the blurb about how to not get it again on my laptop at some point.’ [Participant 16, Male, age 22]*

*‘The testing…I would do it with a group of my mates, probably at a public space, like a coffee shop or something. But I would be at home for the results–so I would access the questionnaire [medical consultation] from my laptop.’ [Participant 2, Male, age 20]*

Participants also highlighted that they estimated that their interaction with the system would be in short chunks of time, possibly reflecting the perceived challenges of mobile access (both technological, e.g. signal drops, and social, e.g. interruption).

*‘I wouldn’t mind filling in the questionnaire while I am out and about, but if this is a web app, I would be concerned I might lose Internet connection and lose everything so I would probably wait until I got home’[Participant 6, Male, age 19]*

Opinions were varied when participants were asked whether they would download and install the application or access it over the Internet and responses highlighted some confusion over the differences. The majority of participants highlighted a preference for an application that would require download and installation (native), as opposed to one they would access through their web browser (web).

*‘Web apps aren’t ideal…because… I hardly EVER use web apps over standard apps, because it’s too much …you’ re always ‘inside something else’, like you re inside Safari… and it has its own layer of complexity and options and you kind of looking through Safari to get to something that really wants to be first level.’ [Participant 21, Male, age 24]*

Nevertheless participants highlighted a one-off context of use, with the intention to delete the app after they have got their results and treatment.

*‘I prefer web apps…I don’t like to download apps as it clogs up my phone, so having a web app means you can go to it without having downloaded it… I am not sure how many times I would use this app, so it would just get deleted… but obviously in terms of style and aesthetics, there are limitations to web apps.’ [Participant 18, Male, age 22]*

Participants’ preferences regarding the various features of the app, such as testing device connection, SMS-based partner notification, and e-prescription format, were also varied.

## Discussion

This paper explored user requirements for a novel mobile-based sexual healthcare intervention. Review of past research showed that while some consideration has been given to user interface design features for mobile health apps for previously diagnosed chronic conditions, little is known about user preferences for applications which can deliver STI test results and manage treatment of a new diagnosis. An exploratory research approach, using focus groups involving 49 young people, was therefore followed and led to a characterisation of user requirements under a number of themes.

The results of the current study suggested that privacy was a major issue for potential users of this kind of system. While privacy has been discussed in the literature on web and mobile based systems [5], much of the focus has been on the potential for sensitive data to be shared over the network and a consequent concern regarding institutional policies for treatment of data and its security [41, 42]. Nevertheless, these studies highlight young people’s concerns with privacy. In the current study, the participants’ primary concern was much more around social, proximal aspects of privacy, emphasising the risk of being overlooked or others seeing messages appear on their phone.

This suggests that design of mobile applications for sensitive healthcare applications needs to carefully consider what is visible and accessible on the patients’ mobile phone. We therefore suggest that the application should be password protected with a timeout facility so that users will need to login again after a period of inactivity. We also propose that app-specific privacy settings (in addition to what is provided automatically by their mobile operator) should be available. This could be in the form of a dashboard and settings which should span across access modalities (mobile/ desktop), system notification modalities, automatic keypad, lock geo-location tracking, screen lock for idle timeout, ad targeting, etc. The design of the application itself should also be discreet so that it not obvious to any observer that a user is interacting with a sexual health interface (due to the stigma associated with this [43]. This might include the use of symbol-based, non-descript logos and app icons, use of ‘ambiguous’ terminology for the name of the app and ‘subtle’ language and nomenclature. Discreet design should also aim to avoid incorporating client-identifying data into the interface whenever possible (for example avoid including the user’s personal details on screens).

While users were less concerned with institutional privacy and security issues than we might have expected, they did still raise some issues related to this, so it is also important to address these concerns within the design of appropriate applications. Recommended features would encompass the inclusion of confidentiality and security policies, just in time disclosure before the app is allowed to access sensitive information (such as location) and assurances (for example through the use of question mark icons) explaining the need for the system to ask for sensitive information.

A second major theme to emerge from the focus group discussion was around the credibility and legitimacy of the service and trust was mentioned frequently in relation to these concerns. The results align with previous research which suggests two main dimensions of credibility: trustworthiness (or belief in the integrity of the provider) and expertise (perceived competence of the provider) [44]. Credibility has been described in the literature in terms of “initial judgments based on surface traits” [45] and in the context of an online interaction, which is predominantly nonverbal, a number of authors have argued that it is based on the ‘look and feel’, aesthetics or design of an interface [46–48]. The focus group findings support this view to some extent, but also highlighted additional features which could impact credibility judgments, for example wide adoption of the service and social media presence would encourage users to try the system. These sub-themes seem similar to Kamthan’s concept of ‘reputed’ credibility which refers to the influence of references to third parties as a means of generating what he calls ‘passive’ credibility [48]. On the basis of the focus group findings we suggest that the design of healthcare applications should aim to provide both explicit and implicit credibility cues, both of which will be expected play a role in users’ judgments. Examples of explicit cues include clearly identifying the service provider (for example by including an ‘about us’ section to highlight the legitimacy of the healthcare service), including affiliations to trusted organisations (for example through including visual logos of affiliated healthcare organisations) and providing assurances of medical content accuracy (for example through links to trusted resources and reference to adherence to established medical guidelines). As suggested by focus group discussions, implicit credibility cues can be provided through the design of the application, both in terms of the ‘look and feel’ of the interface and the language used. Additional implicit credibility cues can be provided by features that demonstrate that others are using the system effectively, for example via a social media presence and user reviews.

The next issue to emerge from discussions was around the clarity of the user journey. This encompassed users’ understanding what to do at any given stage of the interaction (with a particular concern for avoiding making errors), but also where they were in the wider process and also how their interaction fitted within the wider healthcare system including what support was available. This implies that design should firstly aim to support users by making it clear what information they need to input at any stage and help them to avoid errors. Where the app includes a decision support system (such as a medical consultation to decide if it is safe to prescribe online) the questions should be relevant and dynamic, using logic to filter out questions based on the information already provided by users. Error support should encompass not only by providing meaningful error messages that provide clear information on how to recover when mistakes are made, but also proactively indicating once an acceptable value has been entered within a field. Users should also be reassured that there are no catastrophic sequences for having errors when completing the medical consultation form. Users should be notified that they will be given opportunities to change or rectify information they have provided before the end of the process. These recommendations align with general usability guidelines and heuristics which emphasis support for error recovery [49, 50]. On the basis of the focus group findings we also suggest that the design should help untangle the complexity of each healthcare journey without trivialising or over-simplifying the clinical context. Where possible graphical representations of ‘progress made’ should be provided for multi-page forms, displaying how many steps have been completed and how many are left. An overview of the content at the start of a task (e.g. online medical consultation) should also be considered to help the user understand the sub-tasks involved in completing the whole interaction. Attention also needs to be paid to supporting the user in terms of their understanding of the mobile app within the wider context of the healthcare system. Where possible, providers should consider various possibilities for providing support to users as well as provide flexibility in the delivery of services. While mobile technologies could be beneficial, it is also worth allowing for alternative methods of delivery support such as through the provision of ‘offline’ means of care, (through health professional-staffed helplines or live help-chats) or through the availability of making contact with healthcare services for a face-to-face session. The provision of seamless transitions between online and offline mode of healthcare delivery should also be considered, although we recognise this might be subject to interoperability and infrastructures challenges.

The final theme to emerge from the discussions was around the three-way fit between the technology, the task and the context of use. Task-technology fit has been identified in previous literature and refers to the mapping between the characteristics of a technological solution and the characteristics of the task to be performed [51]. While participants discussed a number of issues which fit broadly within this definition, these also overlapped with the added dimension of fit with the context of use. We therefore identify the theme of task-technology-context fit to encompass these findings. The findings suggest that design should accommodate both ubiquity and mobility. In terms of ubiquity, design should accommodate the different contexts of use of users; users should be able to access the service from a variety of platforms, mobile devices and operating systems. Platform and device independence should also be complemented by the provision of a seamless switch between contexts of use. Particular emphasis should be placed on specifically supporting the mobile context of use. Users should be able to save their interaction with the app and not lose their progress and particular effort should be made to ensure ‘short bursts’ of interaction can be accommodated to overcome possible problems due to limited bandwidth requirements or interrupted transaction and communication. There were also considerable inter-participant differences in terms of preferences, suggesting perhaps that the theme could be extended to encompass the four way fit between person, technology, task and context. The technology choice findings suggest that a ‘one size fits all’ approach to design may not always be appropriate and that some customization may need to be offered to meet user requirements. However, there seems to be a design tension between the need for user customisation and the ‘one-off, fast interaction’ context of use of the app as described by the participants. Good design of defaults is essential to err on the side of caution and provide a balanced solution between zero-configuration and counter-productive personalisation.

Overall, the findings of the focus groups led us to propose a number of user interface recommendations specific to the design of a mobile system for remote management of sexually transmitted infections. These are summarised in Table 2. Design guidelines play an important role in Human-Computer Interaction (HCI) as they can potentially assist the design of future systems, first by guiding the design itself and second by providing a set of heuristics that can be used to support usability inspection of developed systems. The recommendations proposed here are intended to supplement more general user interface design guidelines such as Nielsen’s usability heuristics for user interface design, Shneiderman’s Golden Rules of Interface Design and Norman’s Principles of Design [52–55].

**Table 2:** Proposed user interface design recommendations for mobile health applications

Theme	Sub-theme	Design recommendations	Description
Privacy & security	Social privacy	Password protection	App-level password or passcode protection should be implemented every time the user accesses the app or after a certain period of inactivity
		Privacy settings	App-specific privacy settings should be available; default settings should err owards providing higher levels of privacy
		Discreet design	Logos, icons and terminology used should be subtle and not draw attention to sexual health
	Institutional privacy & security	Assurances & disclaimers	Information should be provided on the reason for requesting any sensitive data
		Just-in-time disclosures	Disclosures should be provided before allowing the app to access sensitive content (such as geo-location information) through APIs
		Confidentiality & security policy	A clear policy on how information will be collected and stored should be provided and should be available to view in a number of formats (e.g. online, or download and read offline).
Credibility & Legitimacy	Explicit Credibility	Assurances of medical content accuracy	Apps should provide information supporting their adherence to established medical guidelines including references/links to trustworthy third party material or resources
		Identification of ‘app operator’	Apps should disclose information about the legitimate organisation behind the application, including how to contact them; web apps and online support should use a culturally relevant domain name and support information should be up to date
		Affiliations	Any affiliations with existing respected providers (such as the NHS) should be clearly displayed, for example through the integration of relevant logos within the app design
	Implicit credibility	User community cues	Accompanying website / social media / app store presence should include user reviews and/or case studies
		Visual aesthetics	Culturally relevant and conventional health-related colour schemes and typeface should be used
		Language	The language used should have a serious and professional tone; sentences should be concise and use uncomplicated structures; a glossary of medical terms should be available
User journey support		Simplification of complex healthcare journeys	Provide graphical representation of progress made for multi-step interactions; give overview of steps to be completed at the start of the task
		Content relevance and logic	Where the app includes a decision support system (such as a medical consultation to decide if it is safe to prescribe) the questions should be relevant and dynamic, using logic to filter out irrelevant questions based on the information already provided
		Specific and appropriate feedback	Visual (or audio) cues should be used to indicate erroneous data entry and also proactively indicate once a user has entered acceptable data in a field; error messages should support error recovery
		Reassurances	Take steps to reassure users that there are no catastrophic consequences of making errors in completing an online consultation; provide opportunities to change erroneous inputs
		Flexibility in the delivery of support	Provide flexibility to users in terms of how they can access support (e.g. online and offline; web, telephone and face to face)
Task-technology-context fit		Ubiquity	Design should accommodate different contexts of use, supporting platform independence and the ability to switch seamlessly between contexts of us
		Mobility	Design should support mobile context of use which may include interruptions due to concurrent activity or lack of connectivity; design should thus accommodate short bursts of interaction, allowing user to save interaction with app and not lose progress
		Customisation	Users should be able to customise parameters of the app to accommodate their own preferences, particularly for system notifications

In this paper, we adopt the term ‘design recommendations’ to describe the design insights arising from the analysis. This is in line with conventions within the field of Human Computer Interaction (HCI), [55] where this term is well understood by designers as describing the typical means for propagating human factors knowledge and evidence based recommendations into the development of novel software applications as formative design input [56]. User interface design recommendations, as used in this context, are not intended to carry equivalent weight to clinical guidelines, but instead are intended to provide a practical guide to design.

### Limitations and future work

While the qualitative focus group approach followed in this study has considerable benefits given the exploratory nature of this work, it also brings some limitations. Sample sizes are relatively small and while samples were representative of those at high risk of STIs in terms of age, the use of samples drawn from those currently in education within only two geographical locations means that the findings may not be fully transferable. The authors of this paper also acknowledge that an online clinical care such as this one requires a certain level of literacy and health literacy. It may well be that the higher cognitive skills needed to answer clinical questions online mean that it is not appropriate or medically safe to manage people with learning difficulties and/or people for whom English is not their first language remotely. This is not a limitation of our approach to this study, but more recognition that no matter how medical care is provided, it must be medically safe and appropriate to the individual. We anticipate that people with learning difficulties and impairments would be best managed in clinic where their more complex needs could be met. In addition, the experience prototype used in the focus groups was not fully functional and some of the specific design features illustrated in the prototype may have influenced the direction of the conversations. The application described in this paper is designed to provide clinical sexual health care, the focus of this paper is to examine user interface design features for such a novel e-sexual health intervention for young people. The results are based from data that has been collected by two age groups and further analysis is required to fully explore any gender-based, age or setting differences. Traditional face-to-face services do not employ a gender-specific approach to design of general sexual health services; men and women are seen in the same services by the same clinicians at the same time, although there are exceptions for certain groups such as men who have sex with men. It is unlikely that we would design a separate male & female interface although the clinical questions would clearly be different. The recommendations drawn from this work should therefore be considered tentative at this stage and further work is needed to enhance the transferability of our findings and validate their usefulness in practice. In the next stage of our work the findings of this focus group study are feeding into the design of a working application, allowing users to access STI (in this case Chlamydia) test results on their phone, complete an online clinical assessment and receive access to treatment (if they are safe to treat). This will be tested with patients in a clinical setting, providing a wider evidence base on which to assess the extent to which the recommendations proposed here impact on usability and user acceptance in practice. Furthermore, In line with the HON criteria for medical apps [56], we anticipate annual review of the content of this application by clinicians. This would enable changes in clinical guidance (e.g. in terms of choice of antibiotic or changes in other elements of care to be incorporated as needed in a timely manner. This is entirely feasible and mirrors regular clinical updating of clinical protocols and procedures within a service.

## Conclusions

The work reported here reflects a multidisciplinary collaborative effort involving HCI practitioners, health professionals and key stakeholders. We argue that designing mobile applications to successfully integrate with healthcare practice is complex and will benefit from bringing together expertise from technological, social and medical perspectives. This work paves the way for a greater focus on user-centred approaches for mobile health interventions.

While the recommendations discussed here were based on consideration of a specific sexual health intervention (the eSTI^2^ application) we envisage that a number of the design insights identified might also be applicable to the design of mobile health apps in general and further work could explore the extent to which they generalise to other settings.

## Additional files

Additional file 1:
**Evidence based mobile health application design principles.** Summary of existing mobile health application design principles and features. (DOCX 14 kb) Additional file 2:
**Topic guide for the focus group discussions.** This is the topic guide that was adopted to structure and guide the focus group discussions. (DOCX 23 kb) Additional file 3:
**Design scenario for partner notification.** This is an example of the scenarios used in the focus group discussions, and it involves a person (Sharon) and a narrative about her user journey with the application. (DOCX 13 kb)

## Abbreviations

STI: Sexually transmitted infection
HCI: Human computer interaction
FE: Further education
HE: Higher education
APP: Application
