# Supplementary material for: User interface design for mobile-based sexual health interventions for young people: Design recommendations from a qualitative study on an online Chlamydia clinical care pathway
Source: BMC Med Inform Decis Mak. 2015 Aug 26;15:72. doi: 10.1186/s12911-015-0197-8 (PMC4549868; doi:10.1186/s12911-015-0197-8)
Supplement: Additional file 1: — Evidence based mobile health application design principles. Summary of existing mobile health application design principles and features. (DOCX 14 kb) [file 12911_2015_197_MOESM1_ESM.docx]

**Appendix 1**

**Table I:** Evidence-based mobile health application design principles

|  | **Study** | **Features/Design Principles** |
| --- | --- | --- |
| Sexual Health Specific | Mobile health applications for self-monitoring and self-management of HIV [17] | -Customisation of reminders  -Automated and location-based reminders for medication adherence  -Password protection  -Data share with healthcare professionals |
|  | SMS-based intervention for HIV treatment adherence [19] | -SMS reminders, incentives and signs of ‘affection’ |
| Non Sexual Health Specific | Mobile health applications to assist patients with diabetes [21] | -Automatic data transfer  -Motivational UI  -Health benefits in relation to effort required  -Dynamic usage  -Context-sensitivity |
|  | Mobile health application for diabetes [20] | -Communication with primary care providers |
|  | Mobile health app for the adherence to cancer treatment. [22] | -Daily symptoms diary |
