# Supplementary material for: User interface design for mobile-based sexual health interventions for young people: Design recommendations from a qualitative study on an online Chlamydia clinical care pathway
Source: BMC Med Inform Decis Mak. 2015 Aug 26;15:72. doi: 10.1186/s12911-015-0197-8 (PMC4549868; doi:10.1186/s12911-015-0197-8)
Supplement: Additional file 2: — Topic guide for the focus group discussions. This is the topic guide that was adopted to structure and guide the focus group discussions. (DOCX 23 kb) [file 12911_2015_197_MOESM2_ESM.docx]

**Appendix 2**

| Scenario 6 - Partner Notification | Design Features Associated |
| --- | --- |
| Sharon’s results were positive so she completes the online medical consultation in order to receive her prescription electronically.  As she successfully completes the questionnaire, she realises that she will need to notify her partners. Sharon has been living with her current boyfriend Tom for 2 months, but in the past six months she has had 4 different partners. | Accessing an online partner notification system  Notifying partners directly  Notifying partners indirectly  Notifying partners via SMS  Notifying partners face to face  Notifying partners via email |
