# Supplementary material for: User interface design for mobile-based sexual health interventions for young people: Design recommendations from a qualitative study on an online Chlamydia clinical care pathway
Source: BMC Med Inform Decis Mak. 2015 Aug 26;15:72. doi: 10.1186/s12911-015-0197-8 (PMC4549868; doi:10.1186/s12911-015-0197-8)
Supplement: Additional file 3: — Design scenario for partner notification. This is an example of the scenarios used in the focus group discussions, and it involves a person (Sharon) and a narrative about her user journey with the application. (DOCX 13 kb) [file 12911_2015_197_MOESM3_ESM.docx]

**Appendix 3**

**Topic Guide**

| **Topic** | **Guide** | **Details** | **Probes** |
| --- | --- | --- | --- |
| **Connectivity** | Preferences on ‘accessing’ the esti² HTI | User preferences for accessing the system |  |
| **Registration** | Preferences on registration mechanisms  Preference on confirmation of registration  Preferences on visual design/layout/content of registration page. | User views on confirmation of registration with the service? Tone/wording of message  User preferences for the registration page visual layout | Mock-up of registration page |
| **Receiving Test Results** | Preferences on receiving test results?  Preferences of setting reminders for failure to access results  Preferences on visual design/layout/content of login page | User preferences on receiving test results  Tone/wording of message  User views on registration page design | Mock-up of registration page with design alternatives.  Example of SMS to access results. |
| **Accessing Results** | Preferences on data collection required prior to accessing results  Preferences on negative results page.  Preferences on positive results page. | Users’ views on data collection items data items and mechanisms  Users’ views on negative/positive results page | Mock-up of data collection screen with all fields.  Mock-up of results page. |
| **Clinical Assessment** | Preferences on online prescription page. | Users’ views on the questions for the clinical assessment (quantity, input method, confidentiality concerns.) | Mock-up of clinical assessment |
| **Electronic prescription** | Preferences on e-prescription  Preferences on locating nearest participating pharmacy | Users’ views on how they would receive e-prescription  Users’ preferences of collecting prescription  Users’ views on mechanisms to identify nearest pharmacy | Mock-up of QR code on screen page.  Mock-up of pharmacy locator |
| **Partner notification** | Preferences on partner notification mechanism | Users’ views on partner notification stage before prescription?  Users’ views on content of anonymous message to partner | Mock up on prescription page  Mock-up of partner notification SMS |
